# Fourier-Transform-Based Surface Measurement and Reconstruction of Human Face Using the Projection of Monochromatic Structured Light

**DOI:** 10.3390/s21072529

**Published:** 2021-04-04

**Authors:** Bingquan Chen, Hongsheng Li, Jun Yue, Peng Shi

**Affiliations:** School of Science, Qingdao University of Technology, Qingdao 266525, China; chen_bingquan@yeah.net (B.C.); lihongsheng1123@qut.edu.cn (H.L.); yuejun@qut.edu.cn (J.Y.)

**Keywords:** pattern recognition, 3D imaging and sensing, structured light, polarization, human face, reconstruction

## Abstract

This work presents a new approach of surface measurement of human face via the combination of the projection of monochromatic structured light, the optical filtering technique, the polarization technique and the Fourier-transform-based image-processing algorithm. The theoretical analyses and experimental results carried out in this study showed that the monochromatic feature of projected fringe pattern generated using our designed laser-beam-based optical system ensures the use of optical filtering technique for removing the effect of background illumination; the linearly-polarized characteristic makes it possible to employ a polarizer for eliminating the noised signal contributed by multiply-scattered photons; and the high-contrast sinusoidal fringes of the projected structured light provide the condition for accurate reconstruction using one-shot measurement based on Fourier transform profilometry. The proposed method with the portable and stable optical setup may have potential applications of indoor medical scan of human face and outdoor facial recognition without strict requirements of a dark environment and a stable object being observed.

## 1. Introduction

The measurement and reconstruction of three-dimensional (3D) human face has been a very challenging problem, which has attracted scientists and technologists for developing new methods with higher accuracy and better adaptability, which is necessary and important for the application purpose, such as medical imaging [1,2,3] and facial recognition [4,5].

Methods of 3D facial scan using structured light have been investigated and developed for about three decades. The proposed method of Vázquez and Cuevas for 3D facial reconstruction and classification was based on the use of structured-light projection and the phase-shifting technique [4]. The investigation by Dunn and co-authors showed the feasibility of the imaging system for reconstructing the surface of human body using structured light [6]. The key part of the work by Bhatia et al. was the optical surface scanner with white light to project coded patterns of structured light [7]. Gregory and Lipczynski completed the study of monitoring facial surface based on a structured-light technique, while the measuring system simply consisted of a standard commercial slide projector and CCD cameras [8]. Amor and co-workers presented a face reconstruction method based on a hybrid structured light and stereo sensor technique [9], which featured the combination of the stereo triangulation, data interpolation based on cubic spline models, meshing based on Delaunay triangulation and texture mapping process. Yang et al. employed color structured light to image the skin [10], while this approach may have the same limitations of phase-shifting method using digital-light-processing (DLP) projector. In addition to the basic method based on the combination of phase shifting and coded-pattern projection, Yue and co-authors recently developed their improved registration and graph optimization algorithms [11].

There have been some investigations related to the situations that human body is under moving conditions. Zhang and Huang developed a 3D shape measurement system using digital fringe projections, a high-speed CCD camera and the phase-shifting technique [12]. Kimura et al. aimed at the accurate measurement of the dynamic shape of human foot in motion, while they indicated that the proposed method using fixed characteristic patterns had a requirement that the surface reflectance of the observed object should not be variable [13]. The method of Liberadzki et al. was developed for measuring the human body in motion [14], which employed the projected sinusoidal patterns generated using DLP projector, as discussed by Sitnik [15]. Note that the sinusoidal patterns generated using DLP projector will be affected by the Gamma effect and defocusing issue. The 3D facial reconstruction approach based on one-shot image of the projected structured-light line pattern was recently suggested by Wang [16], while the energy distribution of the projected pattern might be one of the critical issues that should be considered and it would be a problem if background illumination exists.

3D facial reconstruction can also be realized based on laser line scanning using a laser line projector [17], which is the basis for laser 3D slit scanner. Measurements using laser line scanning require that a single line of points is captured at each time, and it is necessary to move either the scanner or the object to acquire additional lines of points for completing the 3D reconstructions of observed objects. Recently, Piedra-Cascón et al. [1], Zhao et al. [2] and Amornvit and Sanohkan [3] compared different facial scanners, and from their conclusions we could see that both slit scanners and structured-lighting techniques are ill-suited for scanning dynamic scenes.

For the existing methods including the commercial technologies, such as the 3dMD Face system (3dMD, Atlanta, USA) and the FaceScan system (Isravision, Darmstadt, Germany), there are some strict requirements for the targets (or the human participants). The individual participant has to maintain a sitting position together with a stable support for head and neck and a stable mandibular position, while the participant must also keep his/her eyes and lips naturally closed and his/her body relaxed. Usually, repeated scans or multiple images are necessary for those methods, which should mostly be carried out under a dark environment. As reported, it is capable of obtaining the best accuracy within the middle part of the participant’s face, while the deviation of reconstruction exists for the upper and lower parts of the face [2].

Generally speaking, the existing techniques discussed and mentioned above basically suffer the following main drawbacks: (i) 3D facial scan and reconstructions using structured light and phase-shifting technique have a rigorous requirement that the human face remain still during the measuring time. (ii) If the projected pattern is based on the sinusoidal fringe generated by DLP projector, the intensity deviation of the sinusoidal-fringe curve affected by the Gamma effect and defocusing issue will result in reconstruction errors. (iii) For structured-pattern projections with white light, background illumination is mostly not allowed during the measurement, which indicates that outdoor in-situ measurement for facial recognition will be impossible.

Thus, aiming at obtaining a 3D reconstruction of human face with higher accuracy and better adaptability, we propose a method that combines the projection of sinusoidal optical signal, techniques of optical filtering and polarization and Fourier transform profilometry (FTP). Note that the projected sinusoidal optical pattern generated by the designed optical system has the features of being monochromatic, higher fringe contrast, time-invariance and adjustable spatial frequency. The monochromatic feature can be combined with an optical filter to effectively reduce the influence of the environmental noisy light signals, which has been a tough restriction for 3D measurement and reconstruction with background illumination.

## 2. Theoretical Analyses

### 2.1. The Generation and Projection of Sinusoidal Fringe Signal

**Figure 1:**
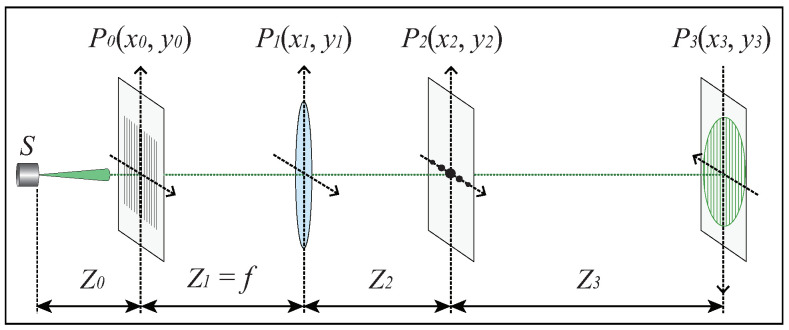
Sketch of the sinusoidal optical signal generator.

Profilometry based on either Fourier transform or phase shifting relies on the projection of sinusoidal fringe patterns. Moreover, the widely-used sinusoidal pattern generated using DLP projector is featured with having the limitations resulted from the requirement of precise synchronization, speed limit of measurement and the nonlinear gamma effect. For the purpose of generating monochromatic, high contrast and truly sinusoidal fringe patterns, we designed and developed the laser-beam-based optical system sketched in Figure 1. The fundamental components of the sinusoidal optical signal generator include a laser source with the wavelength λ=532 nm, a rectangular grating positioned at plane $P_{0}$, a Fourier-transform positive lens with focal length *f* and an adjustable spatial-frequency filter located at plane $P_{2}$.

We now present a theoretical description of the generation and propagation of the sinusoidal fringe pattern based on the designed optical system. The grating at plane $P_{0}$ is illuminated by the optical field from point laser source, as indicated in Figure 1. The optical field right behind the grating, $U_{0}\left(x_{0},\;y_{0}\right)$, is given by

(1) $$\begin{array}{c} U_{0}\left(x_{0},\;y_{0}\right)=\frac{1}{Z_{0}}e^{ikZ_{0}}e^{i\frac{k}{2Z_{0}}\left(x_{0}^{2}+y_{0}^{2}\right)}t_{0}\left(x_{0},\;y_{0}\right), \end{array}$$

where $Z_{0}$ is the distance between the laser source and grating and $t_{0}\left(x_{0},\;y_{0}\right)$ is the transmission function of the grating. $t_{0}\left(x_{0},\;y_{0}\right)$ has the form

$$\begin{array}{c} t_{0}\left(x_{0},\;y_{0}\right)=t_{0}\left(x_{0}\right)=\left[\mathrm{rect}\left(\frac{x_{0}}{a}\right)*\frac{1}{d}\mathrm{comb}\left(\frac{x_{0}}{d}\right)\right]\mathrm{circ}\left(\frac{2|x_{0}|}{H}\right), \end{array}$$

where $x_{0}$ and $y_{0}$ are the coordinate variables of $P_{0}$ and *a* and *d* are the optical parameters of the grating. *H* is the grating width, which is assumed, for the sake of simplicity, to be the diameter of the illumination spot of laser. Since the propagation of field $U_{0}\left(x_{0},\;y_{0}\right)$ from plane $P_{0}$ to plane $P_{1}$ is within Fresnel region, the field $U_{1}^{\prime }\left(x_{1},\;y_{1}\right)$ in front of the lens at plane $P_{1}$ can be written as

(2) $$\begin{array}{c} U_{1}^{\prime }\left(x_{1},\;y_{1}\right)=C_{1}\int_{\Sigma_{0}}U_{0}\left(x_{0},\;y_{0}\right)e^{i\frac{k}{2Z_{1}}\left[{\left(x_{1}-x_{0}\right)}^{2}+{\left(y_{1}-y_{0}\right)}^{2}\right)]}dx_{0}dy_{0}, \end{array}$$

where $C_{1}=\frac{1}{i\lambda Z_{1}}e^{ikZ_{1}}$ is a complex constant and $Z_{1}$ is the distance between the planes $P_{0}$ and $P_{1}$. The field $U_{1}\left(x_{1},\;y_{1}\right)$ after the lens at plane $P_{1}$ should be in the form

(3) $$\begin{array}{c} U_{1}\left(x_{1},\;y_{1}\right)=U_{1}^{\prime }\left(x_{1},\;y_{1}\right)t_{L}\left(x_{1},\;y_{1}\right)=U_{1}^{\prime }\left(x_{1},\;y_{1}\right)e^{-i\frac{k}{2f}\left(x_{1}^{2}+y_{1}^{2}\right)}, \end{array}$$

where $t_{L}\left(x_{1},\;y_{1}\right)=e^{-i\frac{k}{2f}\left(x_{1}^{2}+y_{1}^{2}\right)}$ denotes the transmission function of the lens.

The propagating field $U_{1}\left(x_{1},\;y_{1}\right)$ at plane $P_{1}$ to $U_{2}^{\prime }\left(x_{2},\;y_{2}\right)$ at plane $P_{2}$ can be accurately computed using Fresnel diffraction. $U_{2}^{\prime }\left(x_{2},\;y_{2}\right)$ denotes the field in front of the spatial-frequency filter *F* and is given by

(4) $$\begin{array}{c} U_{2}^{\prime }\left(x_{2},\;y_{2}\right)=C_{2}\int_{\Sigma_{1}}U_{1}\left(x_{1},\;y_{1}\right)e^{i\frac{k}{2Z_{2}}\left[{\left(x_{2}-x_{1}\right)}^{2}+{\left(y_{2}-y_{1}\right)}^{2}\right)]}dx_{1}dy_{1}, \end{array}$$

where $C_{2}=\frac{1}{i\lambda Z_{2}}e^{ikZ_{2}}$ is another complex constant and $Z_{2}$ is the distance between the planes $P_{1}$ and $P_{2}$. The combination of Equations (1)–(4) as well as the expressions of $t_{0}\left(x_{0},\;y_{0}\right)$ and $t_{L}\left(x_{1},\;y_{1}\right)$ can yield that

(5) $$\begin{array}{c} U_{2}^{\prime }\left(\omega \right)=\frac{aC_{3}}{d}\sum_{m=-\infty }^{\infty }\mathrm{sinc}\left(\frac{am}{d}\right)\frac{J_{1}\left[\pi H\left(\omega -m/d\right)\right]}{\omega -m/d}, \end{array}$$

where $J_{1}\left(\cdot \right)$ is the Bessel function of the first kind, $\omega =2\pi f_{x}$ is the spatial frequency $f_{x}$ at plane $P_{2}$ defined by $f_{x}=\frac{x_{2}}{\lambda f}$ and $C_{3}$ is a complex constant given by

$$\begin{array}{c} C_{3}=\frac{1}{\lambda fZ_{0}}e^{i[k\left(Z_{0}+Z_{1}+Z_{2}\right)-\pi /2]}. \end{array}$$

At plane $P_{2}$, an adjustable spatial-frequency filter is specially-designed and employed to select the ±mth-order spectrum described in Equation (5) and allows them to pass through it. Here, we take m=1. We then get the field $U_{2}\left(x_{2},\;y_{2}\right)$ right behind the spatial-frequency filter in the following form

(6) $$\begin{array}{c} U_{2}\left(x_{2},\;y_{2}\right)=U_{2}\left(\omega \right)=U_{2}^{\prime }\left(\omega \right){|}_{m=\pm 1}=\frac{aC_{1}}{d}\mathrm{sinc}\left(\frac{a}{d}\right)\\ \times \{\frac{J_{1}\left[\pi H\left(\omega -1/d\right)\right]}{\omega -1/d}+\frac{J_{1}\left[\pi H\left(\omega +1/d\right)\right]}{\omega +1/d}\}. \end{array}$$

Note that the propagation of field $U_{2}\left(x_{2},\;y_{2}\right)$ from plane $P_{2}$ to $P_{3}$ can also be analyzed using Fresnel diffraction, which allows the field $U_{3}\left(x_{3},\;y_{3}\right)$ at plane $P_{3}$ to be given by

(7) $$\begin{array}{c} U_{3}\left(x_{3},\;y_{3}\right)=C_{4}\int_{\Sigma_{2}}U_{2}\left(x_{2},\;y_{2}\right)e^{i\frac{k}{2Z_{3}}\left[{\left(x_{3}-x_{2}\right)}^{2}+{\left(y_{3}-y_{2}\right)}^{2}\right)]}dx_{2}dy_{2}, \end{array}$$

where $C_{4}=\frac{1}{i\lambda Z_{3}}e^{ikZ_{3}}$ is also a complex constant and $Z_{3}$ is the distance between the planes $P_{2}$ and $P_{3}$. Considering that the size of $\Sigma_{2}\left(x_{2},\;y_{2}\right)$ (≤4 mm in diameter) is much less than that of $\Sigma_{3}\left(x_{3},\;y_{3}\right)$, i.e., the spot size of the sinusoidal fringe pattern (≥100 mm in diameter), we take an approximation that $\lambda f\left(f_{x}^{2}+f_{y}^{2}\right)\ll 2\left(f_{x}x_{3}+f_{y}y_{3}\right)$ for further derivation of Equation (7). Equation (7) then becomes

(8) $$\begin{array}{ccc} U_{3}\left(x_{3},\;y_{3}\right) & = & C_{5}\int_{\Sigma_{2}}U_{2}\left(f_{x},\;f_{y}\right)e^{i2\pi (f_{x}\frac{fx_{3}}{Z_{3}}+f_{y}\frac{fy_{3}}{Z_{3}})}df_{x}df_{y}\\ & = & C_{5}F^{-1}\left\{U_{2}\left(f_{x},\;f_{y}\right)\right\}, \end{array}$$

where the complex parameter $C_{5}$ is given by

$$\begin{array}{c} C_{5}=\frac{\lambda f^{2}}{Z_{3}}e^{i(kZ_{3}-\pi /2)}e^{i\frac{k}{2Z_{3}}\left(x_{3}^{2}+y_{3}^{2}\right)}. \end{array}$$

Equation (8) indicates that the field at the observation plane $P_{3}$ is an inverse Fourier transform of the field output from the spatial-frequency filter. Combining Equations (6) and (8), the field at the observation plane $P_{3}$ has the form

(9) $$\begin{array}{c} U\left(x_{3}\right)=C_{a}\cdot C_{b}\cdot \mathrm{circ}\left(\frac{2f|x_{3}|}{HZ_{3}}\right)\cdot \cos\left(\frac{2\pi fx_{3}}{dZ_{3}}\right), \end{array}$$

where $C_{1}$ and $C_{2}$ are, respectively, given by

(10) $$\begin{array}{c} C_{a}=\frac{2f}{Z_{0}Z_{3}\pi }\sin\left(\frac{a\pi }{d}\right), \end{array}$$

(11) $$\begin{array}{c} C_{b}=e^{i[k\left(Z_{0}+Z_{1}+Z_{2}+Z_{3}\right)-\pi ]}e^{i\frac{k}{2Z_{3}}\left(x_{3}^{2}+y_{3}^{2}\right)}. \end{array}$$

Note that $C_{a}$ is a real constant for fixed spatial distances $Z_{0}$ and $Z_{3}$ and $|C_{b}|=1$. Equation (9) indicates that: (i) $C_{1}$ represents the term of amplitude. We see that the fringe intensity will decrease as $Z_{3}$ increases since *f* and $Z_{0}$ are parameters with fixed values. (ii) The circ-function in Equation (9) defines the range of the fringe pattern with a circle of radius $\frac{HZ_{3}}{2f}$. (iii) The cosine term in Equation (9) has only one variable $x_{3}$ at the plane $P_{3}$ along $x_{3}$-axis, which indicates that the output of this system is monochromatic, high contrast and truly sinusoidal fringes. The cosine term also includes the information that the fringe width (related to the spatial frequency of the output fringe pattern) will increase as $Z_{3}$ increases, while the projected fringe pattern with adjustable spatial frequency might be useful and important for 3D surface measurement.

### 2.2. Imaging Technique and Reconstruction Based on One-Shot FTP

Started by the work of Takeda and Mutoh about four decades ago [18], FTP has been an active research field focusing on 3D surface measurement [19,20,21,22]. However, applications of FTP have been limited to the situations with ideal conditions, while such necessary conditions may include that the surface of the measuring object must be opaque, the surface is highly reflective and diffuse and there is no background illumination.

The penetration depth of green light into human skin is about 2.5 mm [23,24], which represents that the projected laser-beam-based monochromatic structured light onto the human face undergoes multiple scattering within the subsurface layer. As discussed by Holroyd and Lawrence [25], it might be difficult to carry out 3D shape measurement of human face using optical triangular methods, i.e., the structured-light-illumination methods, due to the subsurface scattering brings uncertainties to the reflection measurements at face surface, since the optical triangular method is simply based on the assumption that the direct reflection takes place at the object surface.

When a fringe pattern of structured light is projected onto the human face, the measured reflectance basically consists of three components, i.e., the intensity distribution of directly-reflected light of the projected signal on the face surface, the exited photons that have experienced a multiple-scattering process within the subsurface of face skin, and the background illumination on the face surface. Generally speaking, only the pure direct reflection can be used for determining the 3D shape of human face. However, the direct reflection is usually quite weak, i.e., only few percent of the incident signal for the measurement of human face, while most of the projected light and background illumination will go through the interface and undergo the multiple scattering process [26].

#### 2.2.1. Removing the Effect of Background Illumination Using an Optical Filter

The component related to background illumination is one important issue for in-situ measurement, which has been one reason that most of previous investigations have carried out experimental study in a dark environment. We use a laser-beam-based monochromatic structured light at 532 nm as the projecting fringe patterns, which makes it possible to employ the optical filtering technique in the process of measurement. Thus, an optical bandpass filter centered at 532 nm with FWHM being 10 nm is employed for removing the effects of background illuminations in the experimental setup of this investigation.

**Figure 2:**
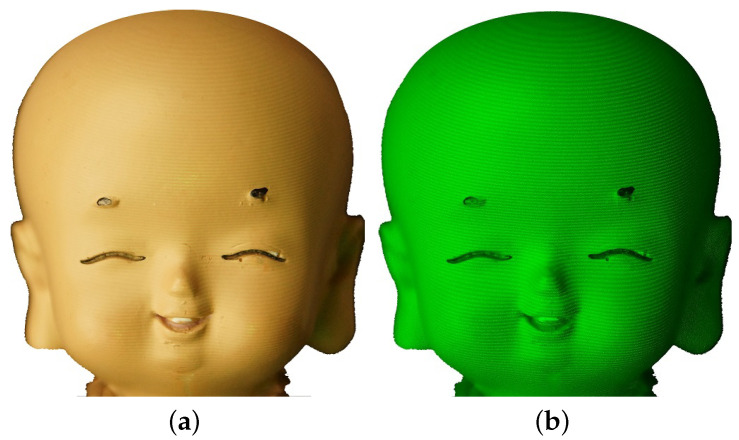
The measuring result of using filtering technique. The target of human head model is illuminated by monochromatic sinusoidal fringe pattern at 532 nm and the background illuminance is E=3365Lux: (**a**) image without filtering; and (**b**) image with an optical bandpass filter centered at 532 nm with FWHM being 10 nm.

As shown in Figure 2, the background illuminance, E=3465Lux, has lowered the fringe contrast and increased the noised signal, and the distorted fringes on the surface of the human head model is almost submerged by the background illumination, as indicated by Figure 2a. However, for the same environmental and experimental conditions, the completed measurement of Figure 2b using optical filtering technique shows greatly-improved fringe contrast with much lower noise level, which ensures the accurate reconstruction of the observed object.

#### 2.2.2. Eliminating the Effect of Multiply-Scattered Photons Using a Polarizer

There have been some efforts towards 3D reconstructions based on the multiply-scattered photons, such as the work by Ohtani et al. [27]. However, the theoretical modeling and computation based on radiative transfer and diffusion theory for the reconstruction of the observed object must be difficult, since it is almost impossible to describe the observed objects such as human face using a one-dimensional model [28].

In our proposed method, the effect of multiply-scattered photons is eliminated by employing polarization technique, which can be ensured since the incident monochromatic structured light and reflected signal from human face or other object surface are linearly polarized, while the signal composing multiply-scattered photons is unpolarized. Thus, a polarizer is mounted on the camera to carry out the measurement of observed object with projected fringe patterns generated using our designed laser-beam-based optical system, as sketched in Figure 1.

**Figure 3:**
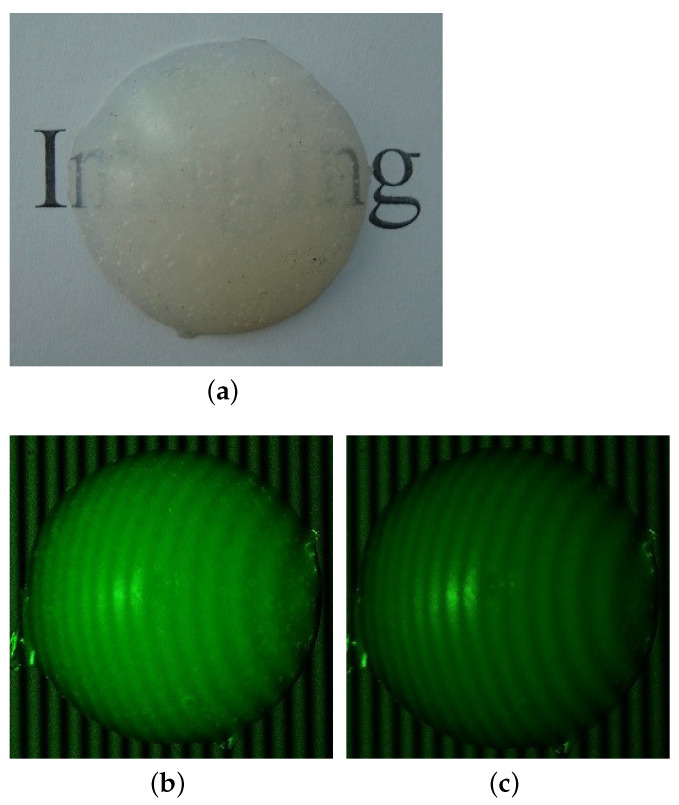
The measuring result of using polarization technique: (**a**) photo of the original target; (**b**) image without polarizer; and (**c**) image with polarizer.

A spherical cap as an observed target, as shown in Figure 3a, is employed for illustrating the use of polarization technique. The spherical cap was made of room-temperature vulcanized silicone rubber using two-component-addition molding. The real part of refractive index of silicone rubber at 532 nm is about $n_{r}=1.4∼1.52$, which is similar to that of human skin [29].

Figure 3b,c shows the measuring results and comparison. The spherical cap was illuminated by monochromatic sinusoidal fringe pattern at 532 nm and the background illuminance was E=0Lux, i.e., a dark environment. Figure 3b is the image without polarizer, while Figure 3c is the measurement with a polarizer mounted on the camera. Obviously, the relative brightness of the image in Figure 3b indicates that the multiply-scattered photons add a strong effect on the surface reflectance, while the effect of multiply-scattered photons depends on the thickness of the silicone rubber and the reflectivity of the bottom surface. In contrast, Figure 3c shows that the effect of multiply-scattered photons has mostly been removed.

#### 2.2.3. Reconstruction Based on One-Shot FTP

**Figure 4:**
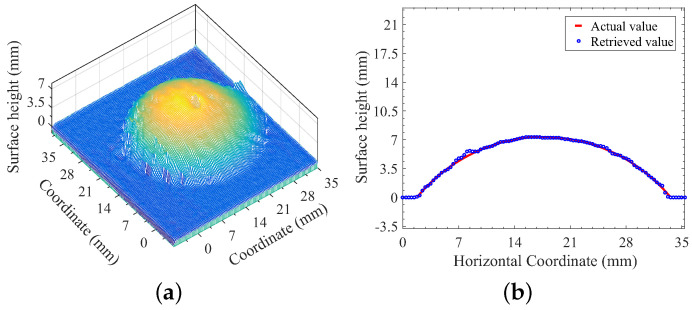
Reconstruction of the spherical cap of vulcanized silicone rubber: (**a**) reconstruction using the measurement of Figure 3c; and (**b**) error of reconstruction.

Note that, if the background illumination and the effect of multiply-scattered photons exist, the facial reconstructions using measurements with structured-light projections would fail or lead to a big error. As discussed above, the quality of the measurement of surface reflectance provides a necessary basis for developing an image-processing algorithm for the retrievals of phase and 3D-surface height of observed object. The image-processing algorithm is based on FTP with a typical triangulation framework of 3D surface measurement, while the general framework of 3D surface measurement can be found in publications by Takeda and Mutoh [18] and Maurel and co-workers [19]. The one-shot FTP-based reconstruction of the measurement of Figure 3c is given in Figure 4a, while the reconstruction error is described in Figure 4b. The reconstruction accuracy based on the comparison between the retrieved curve and the actual shape of the spherical cap, as indicated in Figure 4b, is good and acceptable, and it will not be affected by background illumination if the optical filtering technique is used, as we discussed above.

## 3. Experimental Results of Facial Reconstruction and Discussions

The experimental system basically consisted of two parts: the part of fringe-pattern generator and the part of measurement. The fringe-pattern generator mainly contained the following components: the CW laser source MGL-III-532-200 mW with the wavelength of λ=532 nm and a small divergence angle (≤$1.5^{{}^{\circ}}$), a rectangular grating with spatial frequency of 50 lp/mm, the Fourier transform lens with focal length of 150 mm and a specially-designed spatial-frequency filter that has a V-shape aperture with slit-width of 0.5 mm, which is carved with high precision. The measurement part included a CCD camera, an optical filter centered at 532 nm with FWHM being 10 nm, an optical polarizer, a computer and the image-processing software that was investigated and developed during this study. Note that the cost of each component depends on the the measurement needs and the physical parameters of the individual component. For example, the cost of the CW laser source that we used in this work is about $2000, while it would increase to about $30,000 if we needed 10 times the output power of the CW laser source for potential outdoor measurement and reconstruction with high accuracy.

**Figure 5:**
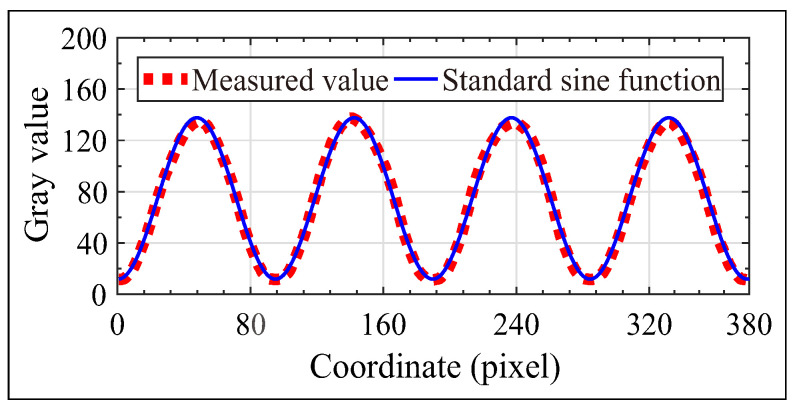
The generated sinusoidal fringe pattern and the comparison of intensity distribution between the generated sinusoidal signal and the standard sine curve.

According to the physical basis of sinusoidal fringe pattern generated in our approach and the FTP-based method discussed above, the reconstruction of human face can be affected by the intensity, contrast and spatial frequency of the projected fringes and the background illuminance. The contrast of the monochromatic fringe pattern generated using the optical system sketched in Figure 1 is approximately equal to unity for a dark background environment, which can be concluded from Equation (9), and was validated using experimental results, as indicated in Figure 5. The light-intensity distribution of the sinusoidal fringe pattern generated using the developed optical system was measured using a CMOS camera with an Aptina CMOS sensor with a size of 5.70 × 4.28 mm and each pixel size of 2.2 × 2.2 μm. It was confirmed that the generated fringe pattern is featured by being monochromatic, time-invariant, high contrast and truly sinusoidal, which is critically important for the measurement and reconstruction of human face in this investigation.

### 3.1. The Effect of Fringe Contrast

**Figure 6:**
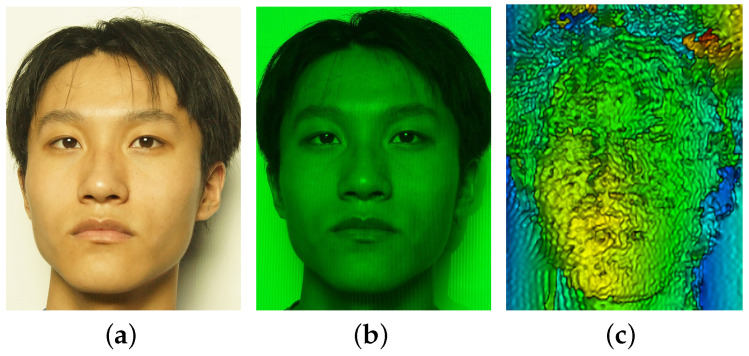
Measurement and reconstruction of a static human face at spatial frequency of 0.3 lp/mm and the background illuminance E=200Lux: (**a**) face image without optical filtering and polarizer; (**b**) face image with optical filtering and polarizer; and (**c**) reconstructed face using the measurement of (**b**).

**Figure 7:**
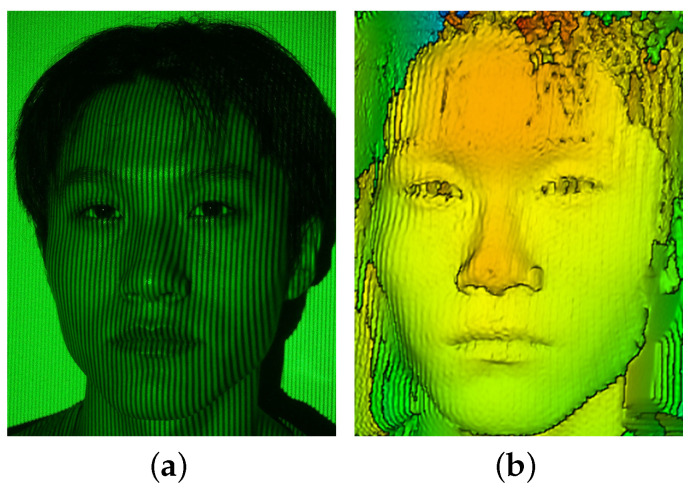
Measurement and reconstruction of a static human face at spatial frequency of 0.3 lp/mm and the background illuminance E=0: (**a**) face image with projected fringe pattern; and (**b**) reconstructed face using the measurement of (**a**).

The first part of our experimental work was on the effect of fringe contrast as well as its relation with the background illuminance, as illustrated in Figure 6. Figure 6 shows the measurement and reconstruction of a human face projected by a sinusoidal fringe pattern generated using our designed optical system, as sketched in Figure 1, while the spatial frequency of the sinusoidal fringe pattern is 0.3 lp/mm and the background illuminance is E=200Lux. The image of Figure 6a was taken without optical filtering and polarizer, and, for the image in Figure 6b, an optical polarizer and an optical bandpass filter centered at 532 nm with FWHM being 10 nm were employed. The fringes on the face in Figure 6b were could be identified, while the fringes were completely buried by the background illumination. The inaccuracy of face reconstruction of Figure 6c from the measurement of Figure 6b is considered as mostly being due to the decreased fringe contrast resulting from the background illumination, which is explained in Figure 7.

The experimental condition shown in Figure 7 was the same as the one in Figure 6 except the background illuminance. The better accuracy of reconstruction shown in Figure 7b compared to Figure 6c indicates that the contrast of the projected fringe pattern is affected by the background illuminance. Note that the fringe contrast of Figure 6b can be increased by increasing the output power of the laser source.

### 3.2. Reconstruction for Both Static and Dynamic Faces at Higher Spatial Frequency

**Figure 8:**
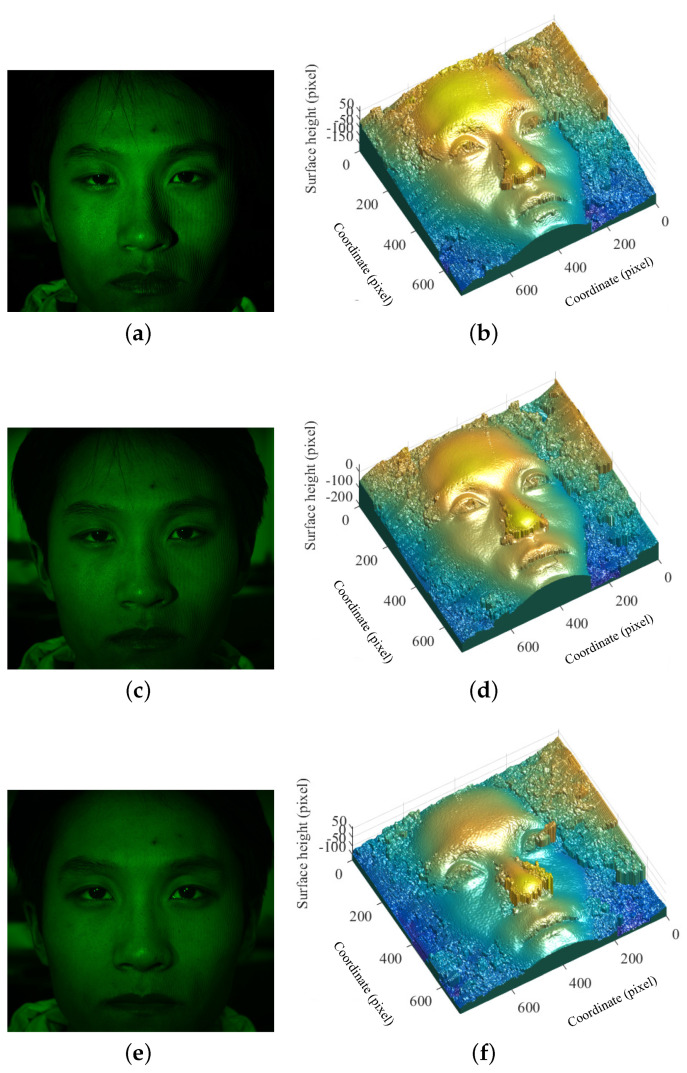
Measurement and reconstruction of a static human face at spatial frequency of 1.0 lp/mm and different background illuminance *E*: (**a**) measurement at E=0; (**b**) reconstruction using (**a**); (**c**) measurement at E=50Lux; (**d**) reconstruction using (**c**); (**e**) measurement at E=175Lux; and (**f**) reconstruction using (**e**).

To optimize the experimental condition, we set the spatial frequency of projected fringe pattern to 1.0 lp/mm and increased the maximum intensity value of the projected fringe by about five times that used in Figure 6 and Figure 7, as shown in Figure 8.

**Figure 9:**
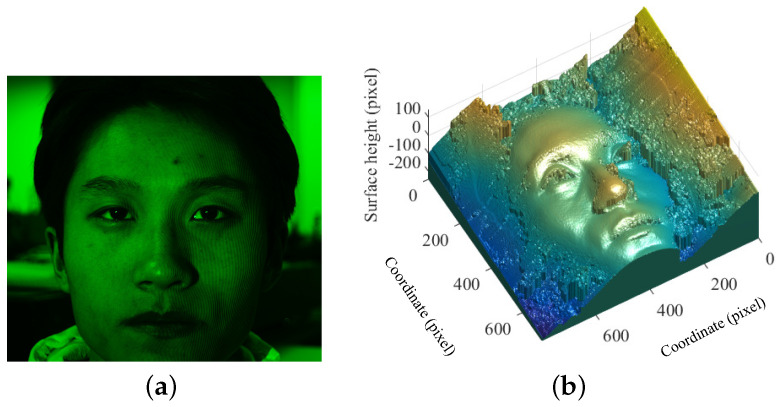
Measurement and reconstruction of a dynamic human face at spatial frequency of 1.0 lp/mm and the background illuminance E=50Lux: (**a**) face image with projected fringe pattern; and (**b**) reconstructed face using the measurement of (**a**).

Different measurements or images, as shown in Figure 8a,c,e, of a static face were taken at the same spatial frequency but different background illuminance. The results presented in Figure 8b,d,f show the same accuracy of reconstruction. With the same experimental condition of Figure 8c, including the position and parameters of the camera, spatial frequency of the projected fringe pattern and the background illuminance, the measurement was completed for a shaking and moving face, as shown in Figure 9a. Obviously, as indicated in Figure 9b, we could also obtain very accurate reconstruction for measuring the 3D surface shape of a moving human face even when background illuminance exists.

The ability to measure a moving human face and obtain an accurate reconstruction is an important feature of this proposed method, which is ensured by all aspects discussed above, mainly including the physical feature and accuracy of the projected fringe pattern generated using the designed laser-beam-based optical system, the optical filtering and polarization techniques and the one-shot FTP-based imaging processing algorithm.

### 3.3. Discussions

The experimental results and corresponding plots are summarized in Table 1 and Table 2.

Compared with the most commonly used approaches, as discussed in the Introduction, the improvements that we made include the following aspects. The first advantage is that the individual participant does not need to be kept in a motionless state, and the proposed method of this work can even obtain high-accuracy reconstruction for a moving participant, as shown in Figure 9b. The second advantage is that our method only needs one-shot measurement for facial reconstruction, while the commonly used approaches usually need repeated scans or multiple images, for which it would also be difficult for a participant to be kept in a stable position for a longer time. The third advantage is that our method is capable of resulting in high-accuracy reconstruction when the background illumination exists, as shown in Figure 8d,f and Figure 9b, while background lighting is not allowed for the most commonly used approaches.

High-contrast and high-quality sinusoidal fringes are critically important for 3D surface profilometry, which was validated by our experiments. Moreover, the spatial frequency and the intensity of the sinusoidal fringe pattern generated using the designed laser-beam-based optical system can easily be controlled and adjusted according to the requirement of measuring different object under different environmental conditions. The optical wave from laser source is monochromatic and generally polarized, thus both the optical filtering and polarization techniques can be used for measurement and image processing, as proposed in our investigation. However, since the optical signal generated by a DLP-based projector is non-monochromatic and unpolarized, it is impossible to extend our approach to methods and applications based on the use of structured light generated by a DLP projector.

Based on the experimental results above, we see that the accuracy of human face reconstruction is basically determined by the intensity, contrast and spatial frequency of the projected fringes and the background illumination. The comparison of the reconstructions of using two spatial frequencies, i.e., 0.3 and 1.0 lp/mm, indicated that the higher spatial frequency may yield better accuracy than the lower one within a validated range. Note that, if the spatial frequency is too high, there will be some problems related to measurement and image processing, which may decrease the accuracy of reconstruction. Thus, the optical parameters including the spatial frequency of projected fringes should be optimized according to the measuring conditions.

## 4. Conclusions

In this study, we carried out both theoretical analysis and experimental investigation for describing a new approach of the measurement and reconstruction of human face. The technical feature of the proposed approach mainly contains three parts: the generation and projection of monochromatic and sinusoidal fringe signal, the imaging technique with optical filtering and polarization and the image processing and reconstruction algorithm based on one-shot Fourier transform profilopmetry. Based on the theoretical analyses and experimental results, the concluding evaluation of the developed method may include the following aspects: (i) The projected sinusoidal fringe pattern generated by the designed laser-beam-based optical system is a monochromatic signal, which is the basis for the use of optical filtering technique for getting rid of the effect of background illumination. (ii) The linearly-polarized characteristic of projected sinusoidal fringe pattern makes it possible to use the polarization technique to effectively subtract the noised signal of multiply-scattered photons coming from the subsurface of face skin. (iii) The sinusoidal fringe pattern also possesses the features of high-contrast, easily-controlled intensity and adjustable spatial frequency, which is capable of resulting in accurate reconstruction of human face using one-shot measurement based on Fourier transform profilometry. Both the optical setup of generating sinusoidal fringe pattern and the measuring equipment such as CCD camera are portable and stable, which helps predict the future applications of accurate medical scan and reconstruction as well as in-situ imaging and recognition of human face in the general indoor and outdoor environments.

## Figures and Tables

**Table 1:** Summary of measurement and reconstruction at spatial frequency being 0.3 lp/mm.

E (Lux)	Using Filter and Polarizer	Measurement	Reconstruction	Accuracy
0	Yes	Figure 7a	Figure 7b	Moderate
200	No	Figure 6a	n/a	Unable to reconstruct
200	Yes	Figure 6b	Figure 6c	Low

**Table 2:** Summary of measurement and reconstruction at spatial frequency being 1.0 lp/mm.

State	E (Lux)	Using Filter and Polarizer	Measurement	Reconstruction	Accuracy
	0	Yes	Figure 8a	Figure 8b	High
Static	50	Yes	Figure 8c	Figure 8d	High
	175	Yes	Figure 8e	Figure 8f	High
Dynamic	50	Yes	Figure 9a	Figure 9b	High
